# Proteomic Analyses of *Acinetobacter baumannii* Clinical Isolates to Identify Drug Resistant Mechanism

**DOI:** 10.3389/fcimb.2021.625430

**Published:** 2021-02-24

**Authors:** Ping Wang, Ren-Qing Li, Lei Wang, Wen-Tao Yang, Qing-Hua Zou, Di Xiao

**Affiliations:** ^1^ Department of Microbiology, School of Basic Medical Sciences, Peking University Health Science Center, Beijing, China; ^2^ Institute for Control of Infectious Diseases and Endemic Diseases, Beijing Center for Disease Prevention and Control, Beijing, China; ^3^ State Key Laboratory of Infectious Disease Prevention and Control, Collaborative Innovation Center for Diagnosis and Treatment of Infectious Diseases, National Institute for Communicable Disease Control and Prevention, Chinese Center for Disease Control and Prevention, Beijing, China

**Keywords:** *Acinetobacter baumannii*, antibiotic resistance, proteomic, TMT, label free, glycopeptides

## Abstract

*Acinetobacter baumannii* is one of the main causes of nosocomial infections. Increasing numbers of multidrug-resistant *Acinetobacter baumannii* cases have been reported in recent years, but its antibiotic resistance mechanism remains unclear. We studied 9 multidrug-resistant (MDR) and 10 drug-susceptible *Acinetobacter baumannii* clinical isolates using Label free, TMT labeling approach and glycoproteomics analysis to identify proteins related to drug resistance. Our results showed that 164 proteins exhibited different expressions between MDR and drug-susceptible isolates. These differential proteins can be classified into six groups: a. proteins related to antibiotic resistance, b. membrane proteins, membrane transporters and proteins related to membrane formation, c. Stress response-related proteins, d. proteins related to gene expression and protein translation, e. metabolism-related proteins, f. proteins with unknown function or other functions containing biofilm formation and virulence. In addition, we verified seven proteins at the transcription level in eight clinical isolates by using quantitative RT-PCR. Results showed that four of the selected proteins have positive correlations with the protein level. This study provided an insight into the mechanism of antibiotic resistance of multidrug-resistant *Acinetobacter baumannii*.

## Highlights

1) 164 proteins exhibited different expressions between drug-resistant isolates and drug-susceptible isolates.
2) Classifying six groups of differential expressed proteins between drug-resistant isolates and drug-susceptible isolates.
3) A specifically expressed polysaccharide on the S21 site of adenylate kinase was found in most MDR strains.

## Introduction

Nosocomial infections caused by multidrug-resistant (MDR) bacteria strains are a serious problem worldwide in decades. *Acinetobacter baumannii* has become one of the most common species that cause nosocomial infections and healthcare-associated infections such as bacteremia, pneumonia, meningitis, skin and wound infections, and urinary tract infection due to its strong biofilm formation ability and the ability to resist nutrient deprivation and antibiotics (Dijkshoorn et al., 2007; Shin and Park, 2015; Mujawar et al., 2019). The traditional first-line treatment of *A. baumannii* uses carbapenem antibiotics such as imipenem, but since the early 1990s, there have been reports of outbreaks caused by imipenem-resistant *A. baumannii* (Tankovic et al., 1994). Other therapeutic antibiotics include aminoglycosides, sulbactam, polymyxin and tigecycline, etc., and combined antibiotics are also being used. However, studies have shown that MDR strains which are resistant to different antibiotics are reported commonly (Li et al., 2006; Peleg et al., 2007; Göttig et al., 2014; Al-Kadmy et al., 2020). Whether for developing new drugs or making full use of old drugs to treat infections caused by MDR strains, it is necessary to fully understand the antibiotic resistance mechanism. A full understanding of the resistance mechanism is critical to improve the eradication rate of *A. baumannii*. Studies have shown that the antibiotics resistant mechanism mainly includes the modification of the target site, inactivation or modification of the drugs by producing enzymes such as β-lactamases, the activation of the efflux pump and the changes of the membrane structure and permeability of bacteria (Dijkshoorn et al., 2007; Zarrilli et al., 2013; Lee et al., 2017; Ramirez et al., 2020).

Genomics and proteomics studies can explore the expression of genes or proteins under various conditions thus to help understand the different mechanisms of bacteria drug resistance. At present, there have been extensive researches on *A. baumannii* through proteomic analysis to explore the relevant mechanisms of drug resistance, drought tolerance, biofilm formation, virulence, and nutrient regulation (Kwon et al., 2009; Shin et al., 2009; Nwugo et al., 2011; Long et al., 2013; Gayoso et al., 2014; Scribano et al., 2019). Researches on drug resistance of *A. baumannii* have studied the differences of a single strain pre and after the induction of resistance (Fernández-Reyes et al., 2009; Hood et al., 2010; Hua et al., 2017) or under different culture conditions (Yun et al., 2011; De Silva et al., 2018), or the difference between susceptible strains and resistant strains (Siroy et al., 2006; Vashist et al., 2010; Li et al., 2015; Wang et al., 2016). However, the researches usually analyze only one or two strains and focused only on a certain antibiotic. Different proteomics methods have their advantages and disadvantages. Using more than one proteomics method can complement each other and enhance the reproducibility and effectiveness of proteomics (Tiwari and Tiwari, 2014). In addition, protein glycosylation is an important means of protein modification, and different glycosylation modifications are critical to protein function (Ahmad Izaham and Scott, 2020). In this study, we intend to use the approach of label free and tandem mass tag (TMT) labeling-based proteomics and glycoproteomes to analyze the different proteins between MDR and drug-susceptible *A. baumannii* to fully clarify the mechanism of the antibiotic resistance.

## Materials and Methods

### Bacteria Strains

Nineteen *A. baumannii* clinical strains (9 MDR and 10 susceptible isolates) were isolated from different patients in the second affiliated hospital of Nanchang University, China, during 2011–2019. The isolates were identified by VITEK-2 compact system and 16S ribosomal DNA identification. The 16S ribosomal DNA were amplified with the primer (16s-PCRF and 16s-PCRR) showed in **Supplementary Table 1**. The fragments were sequenced and blasted in NCBI non-redundant database for species identification. Antibiotic susceptibility of the following antibiotics were tested by Kirby–Bauer test (KB-test): β-lactam antibiotics [piperacillin, ceftazidime, ceftriaxone, cefotaxime, cefepime, imipenem, Unasyn (ampicillin/sulbactam), and Tazocilin (piperacillin/tazobactam)], aminoglycoside (gentamicin and tobramycin), tetracyclines (minocycline and tigecycline), polymyxin B, fluoroquinolone (levofloxacin, Ciprofloxacin), and paediatric compound sulfamethoxazole tablets. Data of these isolates are shown in **Supplementary Table 2**.

### Protein Extraction

**Figure 1:**
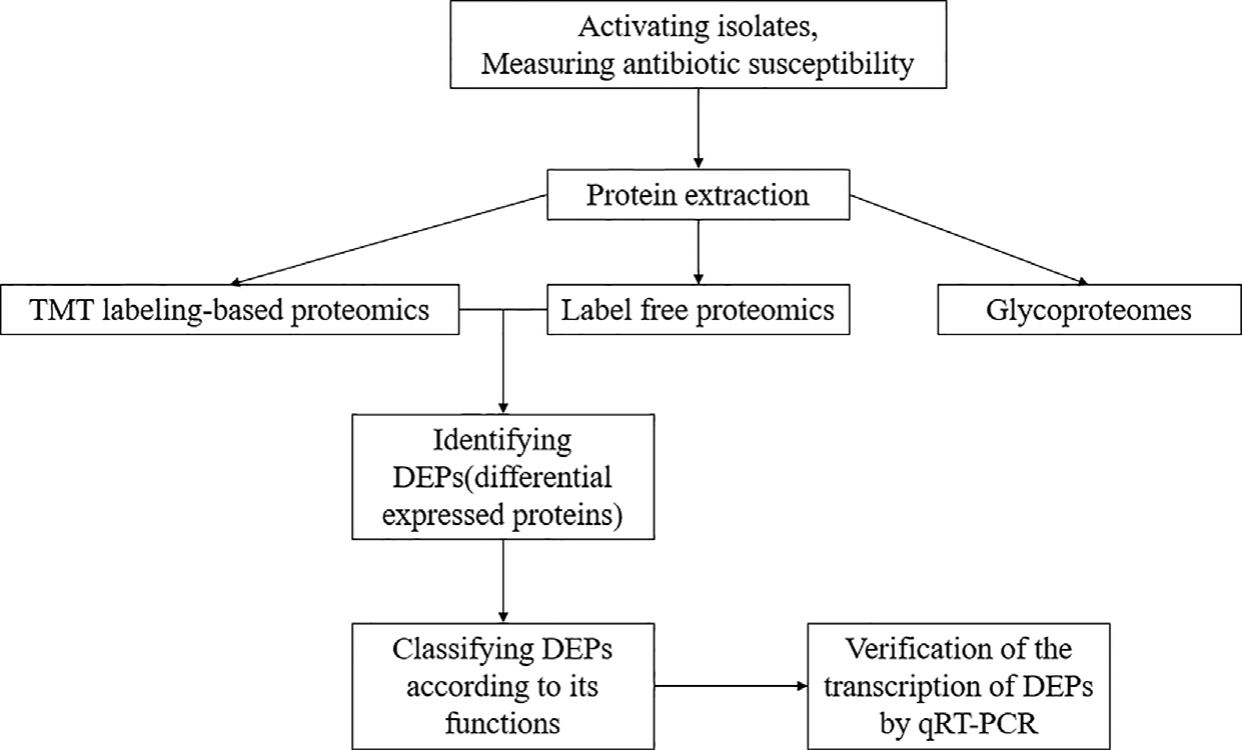
The experiment process of this study.

The experiment process is shown in **Figure 1**. All the isolates were cultured in LB broth at 37°C with shaking until OD_600 nm_ of 0.7–0.8 reached. The cells were collected and lysed by 8M urea in 50 mM triethyl ammonium bicarbonate (TEAB) and ultrasonic breakage for 20 s. The protein samples were collected *via* centrifugation at 16,000×g for 10 min at 4°C. The protein concentration of the supernatant was determined using BCA Protein Assay Kit (Thermo-Fisher Scientific).

### Trypsin Digestions and Peptides Purification

The proteins were reduced by incubation with TCEP (200 mM) at 55°C for 1 h and alkylated by incubation with iodoacetamide (IAA, 375 mM, Thermo Scientific) for 30 min in dark at room temperature. TEAB (100 mM) was used to adjust the urea concentration of less than 1M in all the protein samples, and then the proteins were digested to peptides using trypsin (Promega) at a trypsin/protein ratio of 1:50 (w/w) overnight at 37°C. The generated tryptic peptides were dried by speed vacuum at 4°C and desalted with C18 Spin column. For TMT labeling, the resultant peptide mixture of 10 isolates were labeled using TMT reagent kit (Thermo Scientific, USA) as resistant strain-127 isobaric tag and susceptible strain-126 isobaric tag (Chen et al., 2014). The combinations are as follows: A151 and A9, A159 and A11, A160 and A21, A161 and A90, A162 and A132.

### Nano-HPLC-MS/MS Analysis

The samples were reconstituted in 0.1% formic acid (FA) and separated on a NanoAcquity Ultra Performance Liquid Chromatography (UPLC) system (EASY-nLC 1000, Thermo Scientific). Afterward, the samples were fitted with a nanoAcquity Symmetry C18 trap column (100 μm × 2 cm, NanoViper C18, 5 μm, 100Å) and an analytical column (75 μm × 15 cm, NanoViper C18, 3 μm, 100Å). The mobile phase A was 100:0.1 HPLC grade water/FA, and mobile phase B was 100:0.1 ACN/FA. Each sample was loaded on the trapping column with a flow rate of 2.0 μl/min, followed by separation on the analytical column using a 100 min 3%–35% mobile phase B linear gradient at a flow rate of 0.8 μl/min. Retention Time Calibration Mixture (Thermo Scientific) was used to optimize LC and MS parameters and was used to monitor the stability of the system.

The analytical column was coupled to a high-resolution Q-Exactive Plus mass spectrometer (Thermo Fisher Scientific, San Jose, CA) using a nano-electrospray ion source, which was operated in positive ion mode. The source was operated at 2.0 kV with transfer-capillary temperature maintained at 250°C and S-Lens RF level set at 60. MS spectra were obtained by scanning over the range m/z 350–2000. Mass spectra were acquired in the Orbitrap mass analyzer with 1 microscan per spectrum for both MS and MS/MS. Resolving power for MS and MS/MS were set at 70,000 and 17,500, respectively. Tandem MS data were acquired in parallel with MS, on the top 20 most abundant multiply charged precursors, with higher energy collisional dissociation (HCD) at normalized collision energy of 30V. Precursors were isolated using a 2.0 m/z window and dynamic exclusion of 60 s was enabled during precursor selection. The data were determined twice.

### Proteome Data Analysis

For TMT labeling, Proteome Discoverer (version 1.4, Thermo Scientific, USA) was used to search the UniProtKB/Swiss-Prot database. The parameters were set as follows: integration tolerance, 20 ppm; precursor mass tolerance, 10 ppm; fragment mass tolerance, 0.02 Da. Dynamic modification Oxidation/+15.99 Da and carbamidomethyl/+57.02 Da) were set as dynamic and static modifications. Proteins that were differentially expressed were determined by peptide identifications with 95% confidence interval. Meanwhile, TMT signal analyses showed at least two-fold change in abundance, and its P value was <0.05 in unpaired Student’s t-test.

For Label free, peptide identification and label free relative quantification analysis were carried out using Peaks Studio 8.5 software (Bioinformatics Solutions Inc., Waterloo, ON, Canada). Using *A. baumannii* UniProtKB database (326,258 sequences, downloaded in June 2019). Input parameters: 20 ppm precursor mass tolerance, 0.02 Da fragment mass tolerance. The false discovery rates for protein and peptides were set at a maximum of 1%.

Only those protein groups which passed the filter are displayed in the protein profile heatmap. The relative protein abundance is represented as a heat map of the representative proteins of each protein group. The representative proteins are clustered if they exhibit a similar expression trend across the samples.

### Intact Glycopeptide Enrichment *via* Hydrophilic Interaction Liquid Chromatography (HILIC)

Glycopeptides in the samples were enriched by HILIC (The Nest Group, Inc.). Briefly, the tryptic and desalted peptides were resuspended in 80% ACN. The appropriate amounts of HILIC particle in 80% ACN were placed in Pierce spin columns (Thermo Scientific) and equilibrated three times using 80% ACN, which was followed by sample loading three times and washing two times with 80% ACN. Then, glycopeptides bound to the HILIC column were eluted three times with 100 μl of 0.1% TFA. The samples were dried by a SpeedVac and stored at −80°C until analysis.

The analytical column was coupled to a high-resolution Q-Exactive Plus mass spectrometer (Thermo Fisher Scientific, San Jose, CA) with a nano electrospray ion source operated in positive ion mode. The source was operated at 2.0 kV with the transfer capillary temperature maintained at 250°C and the S-lens RF level set at 60. MS spectra were obtained by scanning over an m/z range of 350–2000. Mass spectra in both MS and MS/MS were acquired in an Orbitrap mass analyzer with 1 microscan per spectrum. The resolving power for MS and MS/MS was set at 70,000 and 17,500, respectively. Tandem MS data on the top 20 most abundant multiply charged precursors were acquired in parallel with MS data, with higher energy collisional dissociation (HCD) at a normalized collision energy of 30 V. Precursors were isolated using a 2.0 m/z window, and dynamic exclusion of 60 s was enabled during precursor selection.

Database searches were performed using Byonic software (v2.13.17, Protein Metrics, Inc.). The following parameters were set for the search: cleavage sites, RK; cleavage side, C-terminal; digestion specificity, fully specific; missed cleavages, 2; precursor mass tolerance, 10 ppm; fragmentation type, QTOF/HCD; fragment mass tolerance, 0.02 Da, and protein false discovery rate (FDR), 1% FDR (or 20 reverse counts). All the other settings were set at their default values.

Byonic scores reflect the absolute quality of the peptide-spectrum match and not the relative quality compared to other candidate peptides. The Byonic score ranges from 0 to approximately 1,000, with 300 being a good score, 400 a very good score, and peptide-spectrum matches with scores over 500 almost certainly correct. The DeltaMod value indicates whether modifications are confidently localized; DeltaMod values over 10 indicate a high likelihood that all modification placements are correct. Therefore, a score over 300, a DeltaMod value over 10, a q-value < 0.05, and an FDR < 0.1% were set as thresholds in this study. Systematic and comprehensive analyses of specific glycopeptides, glycoforms, and glycosylation sites related to our samples from all the proteins identified by Byonic were carried out.

### Bioinformatics Analysis

To further understand the functions of differential expressed proteins (DEP) between drug-resistant and drug-susceptible *A. baumannii* isolates, The DEPs were further submitted to NCBI (National Center for Biotechnology Information) and Uniprot (https://www.uniprot.org/) for GO enrichment analysis (statistically significant differences of GO terms were defined by P < 0.05), KOBAS 2.0 (KEGG Orthology Based Annotation System; http://kobas.cbi.pku.edu.cn/home.do) for KEGG pathway analysis and STRING database (https://string-db.org/) for protein-protein interaction (PPI) analysis. The subcellular localization and the specific information of the DEPs were identified by pSORTb version 3.0.2 (https://www.psort.org/psortb/) and Pubmed, respectively. The potential glycosylation sites were output from NetOGlyc 4.0 Server (http://www.cbs.dtu.dk/services/NetOGlyc/).

### RNA Extraction and Real-Time Quantitative Polymerase Chain Reaction (qRT-PCR)

Eight *A. baumannii* clinical isolates (four resistant strains and four susceptible strains) from the same hospital (data shown in **Supplementary Table 3**) were used to determine the transcription of seven kinds of DEPs: Aminoglycoside (3’) phosphotransferase AphA1 or APH (3’)-Ia (AFV53106), Beta-lactamase AmpC (AFA35105/AFA35107), Outer membrane protein assembly factor BamD (WP_000056810), MFS transporter (RSR57702), ABC transporter (AXV52620), HlyD membrane-fusion protein of T1SS (ENV25944), and Elongation factor Tu (KLT84190). Primers are listed in **Supplementary Table 1**. All isolates were grown overnight at 37°C in LB broth, and sub-cultured 1/100 into fresh LB broth for 4 h. RNA extraction was performed using RNAprep Pure Kit (TianGen). The extracted RNA was reversed to cDNA using the All-in-One™ First-Strand cDNA Synthesis Kit (TaKara). Then qRT-PCR were performed using the 2*SYBR Green qPCR Master Mix (Low Rox). The CT value was obtained by using the 7500 Fast DX instrument, *rpoB* was used as the internal parameter. The normalized relative expression levels of the target genes were calculated by the comparative cycle threshold (2^-ΔΔCT^). The data obtained were analyzed and plotted with Graphpad prism version 5.0. Error bars represent the SDs. Significant differences were defined by P < 0.05 (*), P < 0.01(**), and P < 0.001(***).

## Results and Discussion

Proteomics analysis uses non-targeted research to directly detect the expression of a large number of proteins. In this study, we used TMT labeling-based proteomics, label-free proteomics, and glycoproteomics to analyze the differentially expressed proteins between 9 MDR and 10 drug-susceptible *A.baumannii* isolates. All MDR strains are resistant to Cefepime, imipenem, gentamicin, tobramycin, levofloxacin, ciprofloxacin, and paediatric compound sulfamethoxazole. Drug-susceptible isolates are only non-susceptible to penicillins and cephalosporins of the eight antimicrobial categories listed in **Supplementary Table 2**.

### Analysis of TMT Labeling-Based Proteomics

**Figure 2:**
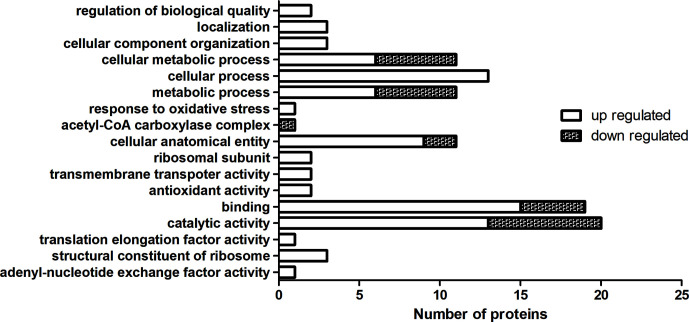
GO enrichment analysis of DEPs in TMT-labeling proteomics. The MDR isolates and drug-susceptible isolates were labelled by 127 and 126 reagent. Up-regulated and down-regulated means 127/126 ≥ 2 and ≤0.5, respectively. Each column represented the number of proteins involved in GO annotation analysis of DEPs in TMT-labeling proteomics. Statistically significant differences of GO terms were defined by P < 0.05.

For the TMT labeling-based proteomics, we randomly selected 10 isolates (5 MDR and 5 drug-susceptible) and made them into 5 drug-resistant and drug-susceptible pairs. Each pair was subjected to two biological replicates. The MDR isolates and drug-susceptible isolates were labelled by 127 and 126 reagent, respectively. In the first pair, 2,270 and 2,730 proteins were found in two biological iterations, and a total of 3,884 proteins were identified. In the second pair, 2,050 and 2,061 proteins were found respectively, and a total of 3,150 proteins were found. In the third pair, 2,695 and 2,200 proteins were found, and a total of 3,782 proteins were found. In the fourth pair, 1,953 and 1,874 proteins were found, and a total of 2,977 proteins were identified. In the fifth pair, 2,021 and 1,927 proteins were found, and a total of 3,051 proteins were found. In order to conduct an overall analysis, we finally selected 127/126 ≥ 2 and ≤0.5 data for analysis. As seen in **Supplementary Table 4**, a total of 70 proteins were obtained with the same expression trend in more than 4 pairs, among which there were 23 kinds of proteins with the same expression trend in 5 pairs. The relative molecular mass of the protein is between 6 and 118 kda, a larger proportion is between 10 and 50kda, and the isoelectric point is between 4.42 and 11.12. 58 up-regulated proteins (127/126 ≥ 2) and 12 down-regulated proteins (127/126 ≤ 0.5) were expressed in MDR isolates. Gene Ontology (GO) analysis can classify genes to different groups according to their functions. Based on the GO annotation analysis, the proteins were classified into three categories: molecular function, cellular component, and biological process. GO analysis with the largest number of proteins involved were shown in **Figure 2**, up-regulated proteins are classified into 20 molecular function related proteins, 11 cellular component related proteins, and 14 biological process related proteins; downregulated proteins are classified into 7 molecular function related proteins, 3 cellular component related proteins, and 5 biological process related proteins. The DEPs of MDR and drug-susceptible isolates focused on catalytic activity and binding. There are 22 KEGG pathways involved in the down-regulated proteins, most of which are involved in metabolic pathways; 39 KEGG pathways are involved in the up-regulated proteins, which are mostly involved in metabolic pathways, carbon metabolism, and biosynthesis of amino acids (**Supplementary Figure 1**). The PPI net showed the interactions of the 59 proteins. The average node degree is 2.78 and the interaction of ribosomal-related proteins is relatively dense (**Supplementary Figure 2**).

### Analysis of Label Free Proteomics

**Figure 3:**
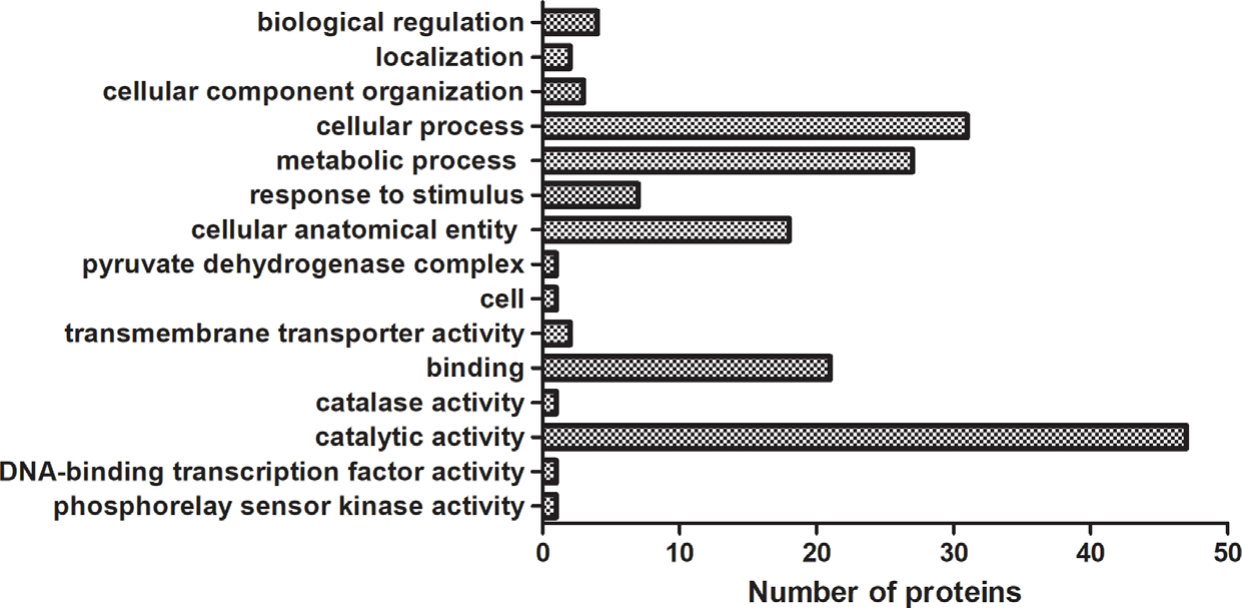
GO enrichment analysis of DEPs in label free proteomics. The proteins in label free proteomics are all present in more than 8 resistant isolates and only in 1 or less susceptible isolate. Each column represented the number of proteins involved in GO annotation analysis of DEPs in label free proteomics. Statistically significant differences of GO terms were defined by P < 0.05.

**Figure 4:**
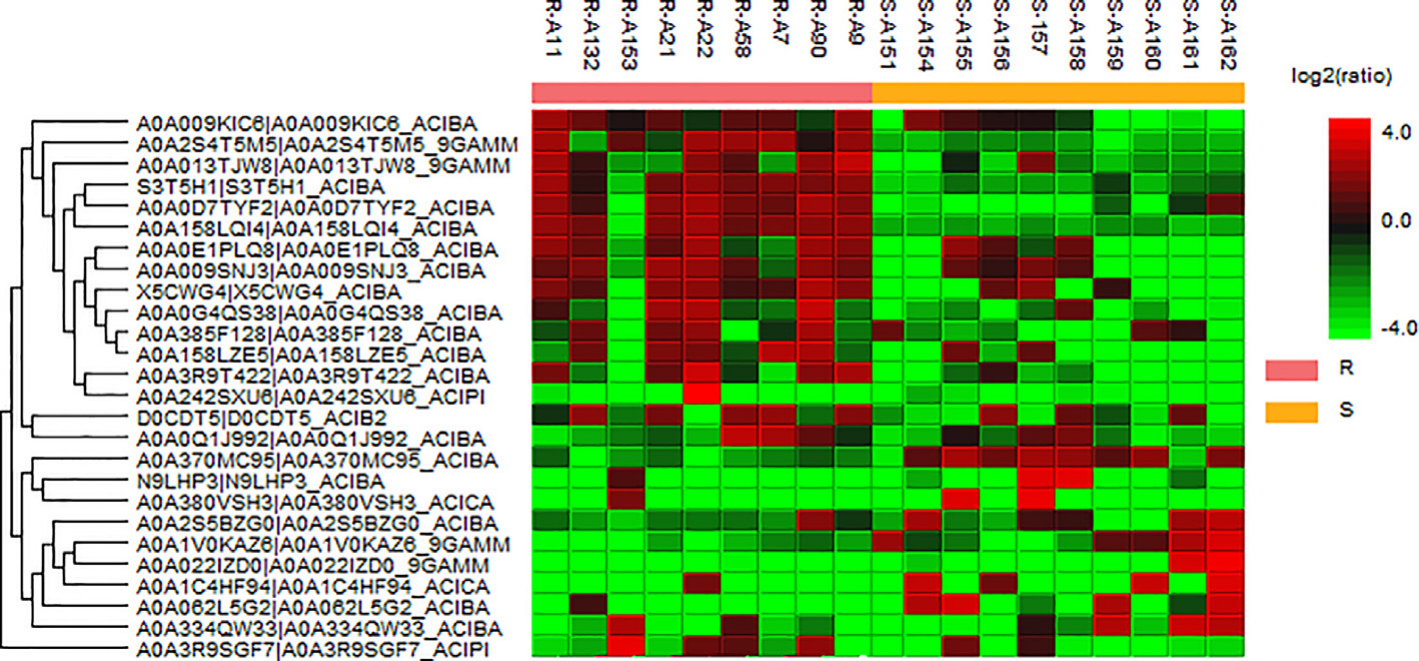
Heat map of the representative proteins of each isolate. “R” means drug-resistant isolates and “S” means drug-susceptible isolates. The representative proteins identified from label free proteomics are clustered if they exhibit a similar expression trend across the samples. The hierarchical clustering is generated using neighbor joining algorithm with a Euclidean distance similarity measurement of the log2 ratios of the abundance of each sample relative to the average abundance.

We analyzed 10 drug-susceptible isolates and 9 MDR isolates by label free proteomics and obtained 102 proteins that are all present in more than 8 resistant isolates and only in 1 or less susceptible isolate (**Supplementary Table 5**). The number of proteins involved in molecular function, cellular component, and biological process are 52, 20, and 33, respectively (**Figure 3**). DEPs mainly focused on catalytic activity, binding, cellular process, metabolic process, and cellular anatomical entity. A total of 48 KEGG pathways (input numbers) were involved in Metabolic pathways (28), Carbon metabolism (11), Biosynthesis of amino acids (10), Glycine, serine and threonine metabolism (7), Valine, leucine and isoleucine degradation (6), Cysteine ​​and methionine metabolism (6), Glyoxylate and dicarboxylate metabolism (5), etc. (**Supplementary Figure 3**). The PPI network diagram showed a total of 87 protein interactions, with an average node degree of 0.437 (**Supplementary Figure 4**). The heat map of similar expressed proteins showed differences in the expression of resistant isolates and susceptible isolates, differences in strains would also lead to different expression levels of some proteins in different bacteria (**Figure 4**).

### Different Expressed Proteins (DEPs) in Drug-Resistant Isolates vs. Drug-Susceptible Isolates

**Figure 5:**
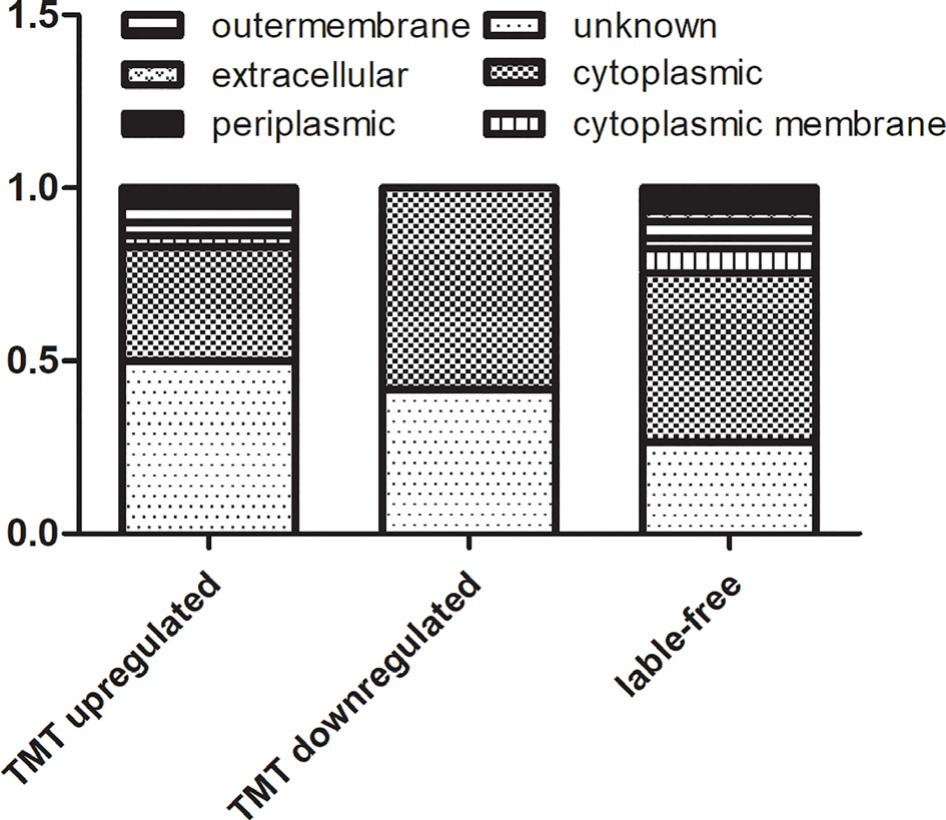
The subcellular localization of DEPs in TMT-labeling proteomics and label free proteomics.

The results from TMT labeling-based proteomics and label free proteomics were combined and we found a total of 164 DEPs. The subcellular localizations of these DEPs were mainly in the cytoplasm, the proteins up-regulated or identified by label free proteomics mainly on the periplasmic or outer membrane of the cell (**Figure 5**). We further classified the proteins into six groups with different functions. According to this classification, there were 12, 20, 22, 14, 60, and 37 differentially expressed proteins of A, B, C, D, E, and F group, respectively. As following:

### Proteins Related to Antibiotic Resistance

In this study, we found 12 proteins more abundant in the MDR isolates classified to group a (**Table 1**). These 12 proteins belong to a variety of known antibiotic resistance proteins, such as beta-lactamases and aminoglycoside phosphotransferase. The resistance of *Acinetobacter* to β-lactam is mainly due to the synthesis and enzymatic degradation of the species-specific β-lactamase, all four types of β-lactamases have been identified in *A. baumannii* (Lee et al., 2017), and the analysis of 23 MDR *A. baumannii* clinical isolates in Taiwan has shown that all *A. baumannii* can encode AmpC cephalosporins (Lin et al., 2011). OXA-23 and OXA-72 belong to class D β-lactamases (Donald et al., 2000; Evans and Amyes, 2014). Metal-dependent hydrolases such as metal β-lactamase (MBL) are zinc-dependent hydrolases that can cleave the β-lactam bond of most β-lactam antibiotics (Parimelzaghan et al., 2016; Kwapien et al., 2017). In this study, both metal-dependent hydrolase-related proteins and β-lactamase-related proteins were up-regulated in MDR isolates. The resistance of *Acinetobacter* to aminoglycoside antibiotics is mainly through N-acetylation, adenylation or O-phosphorylation modification to inactivate aminoglycosides (Seward et al., 1998; Shakil et al., 2008). AphA1b is one of the modifying enzymes involved in aminoglycoside resistance (Nigro et al., 2011). In this study, it is uniquely expressed in MDR isolates, which can explain its prevalence to tobramycin, gentamicin, and other aminoglycoside antibiotics. DacD (D-alanyl-D-alanine carboxypeptidase) belongs to penicillin binding proteins (PBPs) (Spidlova et al., 2018), also called PBP6b in *A. baumannii*, which is involved in the metabolism of peptidoglycans (Cayô et al., 2011). In addition, studies have found that Cell division protein ZapA is related to the resistance of β-lactam antibiotics (Knight et al., 2016). These proteins are all up-regulated in the drug-resistant isolates in this study.

**Table 1:** Proteins related to antibiotic resistance.

Genbank accession	Uniprot accession	Subcellular localization	Protein name	Classification
TPU58603	A0A335ECA6	Unknown	Beta-lactamase OXA-23	TMT up-regulated & Label-free
AJZ68886	A0A0D5W3B6	Unknown	Carbapenem-hydrolyzing beta-lactamase OXA-72 (Fragment)	Label-free
AFI94694	A0A454ATR7	Unknown	D-alanyl-D-alanine carboxypeptidase	Label-free
EEY77424	D0S1V7	Periplasmic	Beta-lactamase	Label-free
ALY01035	A0A0E4HMD5	Periplasmic	Beta-lactamase	Label-free
AFV53106	K4P0R4	Cytoplasmic Membrane	Aminoglycoside (3’) phosphotransferase AphA1 or APH (3’)-Ia	Label-free
EOQ64883	R8Y658	Periplasmic	Beta-lactamase	Label-free
ADX04518	A0A335L319	Unknown	Metal-dependent hydrolase of the aminoacylase-2/carboxypeptidase-Z family	Label-free
VAX45430	A0A0A8XI29	Unknown	Cell division protein ZapA	Label-free
ANA37603	A0A334SNB2	Cytoplasmic	Zn-dependent hydrolase	Label-free
AFA35105	A7Y416	Periplasmic	Beta-lactamase	Label-free
AFA35107	A7Y413	Periplasmic	Beta-lactamase	Label-free

### Membrane Proteins, Membrane Transporters, and Proteins Related to Membrane Formation

The second category contains of 20 DEPs (**Table 2**) including 1 down-regulated protein and 19 up-regulated proteins. Thioredoxin, which is a member of the thioredoxin superfamily, is involved in the virulence of bacteria and also related to the expression of genes related to the ABC transport system (May et al., 2019). This protein is shown to be down-regulated in our results which are consistent with the literature. The upregulated proteins in this group include proteins that play a key role in the formation of the outer membrane, proteins related to maintaining the integrity of the outer membrane, pump proteins, and outer membrane proteins: β Barrel Outer Membrane Protein (BAM) is related to bacterial nutrient acquisition, protein secretion, signal transduction and bacterial survival, and drug resistance (Sikora et al., 2018). Porin has been shown to play an important role in the resistance mechanism (Lee et al., 2017). Lipoprotein can act as a fusion protein to promote the complete biogenesis of the cell membrane (Melly et al., 2019). Studies have found that lipoproteins are upregulated in MDR *A. baumannii* strains (Chopra et al., 2013). SurA is a periplasmic chaperone protein involved in the folding of outer membrane porins, and is closely related to the integrity of the outer membrane (Bell et al., 2018). The alpha/beta hydrolase folding superfamily is a class of hydrolase enzymes involved in lipid metabolism, cell membrane maintenance, virulence, efflux, and metabolism of cell (Johnson, 2017). Outer membrane protein A (OmpA) is the outer membrane protein of bacteria, which is related to the efflux pump and drug resistance of bacteria (Kwon et al., 2017). The expression of OmpW is down-regulated in carbapenem-resistant strains, and its down-regulation can make PBPs unavailable (Tiwari et al., 2012). Study have also found that OmpW has a higher expressed in MDR strains (Chopra et al., 2013). The outer membrane protein CarO is associated with carbapenem drug resistance (Xiao et al., 2016). The multicomponent efflux pump system is widely present in bacteria and it can make bacteria resistant to antibiotics by pumping out antibiotics. Six superfamily of resistant pumps have been identified in *A. baumannii*: major facilitator superfamily (MFS), resistance nodulation division (RND), multidrug and toxic compound extrusion (MATE), small multidrug resistance (SMR), ATP-binding cassette (ABC), and proteobacterial antimicrobial compound efflux (PACE) (Pérez-Varela et al., 2019). In our study, we found that both ABC transporter, MFS transporter, and RND transporter were up-regulated. MFS efflux pump and ABC transporter have been found to be associated with quinolone resistance and beta lactam resistance (Correia et al., 2016; Xiao et al., 2016; Lari et al., 2018; Pérez-Varela et al., 2018). T1SS is formed by HlyB (ABC transporter), HlyD (membrane fusion protein), and TolC (outer membrane). Its C-terminal can carry a secretion signal, and the deletion of the C-terminal will cause secretion blocked (Holland et al., 2016). In our study, both the C-terminal target domain and the hypothetical protein F962_01862 encoding HlyD were up-regulated.

**Table 2:** Proteins related to membrane proteins, membrane transporters, and membrane formation.

Genbank accession	Uniprot accession	Subcellular localization	Protein name	Classification
OIG07664	A0A1S2FR42	Cytoplasmic	Thioredoxin (Fragment)	TMT down-regulated
WP_000056810	A0A009Q2E5	Outer Membrane	Outer membrane protein assembly factor BamD	TMT up-regulated & Label-free
EXC53439	A0A009SK97	Cytoplasmic Membrane	Ubiquinol oxidase subunit 2	TMT up-regulated
CAP01694	B0VSC6	Outer Membrane	Putative lipoprotein	TMT up-regulated
EXC52624	A0A009SI69	Unknown	Chaperone SurA	TMT up-regulated
PZM13340	A0A3F3MK29	Outer Membrane	OmpA family protein	TMT up-regulated
SSU67456	A0A334ZIV7	Unknown	Surface antigen	TMT up-regulated
KCY23168	A0A062IZ25	Outer Membrane	Porin subfamily protein	TMT up-regulated & Label-free
EXC51784	A0A009TK41	Outer Membrane	Peptidoglycan-associated protein	TMT up-regulated
RSR57702	A0A3R9S2V0	Cytoplasmic Membrane	MFS transporter (Fragment)	TMT up-regulated
AXV52620	A0A2P1B3I7	Periplasmic	ABC transporter, phosphonate, periplasmic substrate-binding family protein	TMT up-regulated
EXB00312	A0A009H862	Outer Membrane	OmpW family protein	Label-free
ENV25944	A0A158LU97	Cytoplasmic Membrane	Uncharacterized protein(HlyD membrane-fusion protein of T1SS; cl25633)	Label-free
AFX97596	K7Z0V1	Cytoplasmic Membrane	Kinase sensor (AdeS)	Label-free
ABR18859	A0A2Z5ZA15	Outer Membrane	Carbapenem-associated resistance outer membrane protein (Fragment)	Label-free
SSM96339	A0A333EPN9	Unknown	Putative surface antigen	Label-free
KCY66385	A0A062MBU0	Extracellular	Type I secretion C-terminal target domain protein	Label-free
OTL21614	A0A241YRY7	Cytoplasmic Membrane	Efflux transporter periplasmic adaptor subunit (Fragment)	Label-free
AFI94256	A0A454ASL3	Outer Membrane	Outer membrane protein/peptidoglycan-associated (Lipo) protein	Label-free
WP_024436048	A0A009T321	Outer Membrane	Outer membrane protein assembly factor BamA	Label-free

### Stress Response-Related Proteins

We found there were 22 stress response-related proteins differentially expressed between MDR and drug-susceptible isolates (**Table 3**), among them, the expression of Antibiotic biosynthesis monooxygenase which can oxidize and inactivate antibiotics (Minerdi et al., 2016; Koteva et al., 2018) is interestingly down-regulated. The expression of heat shock proteins and acid shock proteins were upregulated. Heat shock protein is generally used as a molecular chaperone or protease to repair damaged proteins, and its expression increases during stress response such as antibiotic induction. Bacteria with heat shock proteins induced are more resistant to antibiotic environments (Cardoso et al., 2010). Acid shock protein can improve the acid resistance of bacteria (Villarreal et al., 2000). Other proteins related to the stress response and resistance to environment were also upregulated as following: Trigger factor (TF) can play a key role as a molecular chaperone, also related to the resistance to the external environment (Lee et al., 2009). Heavy metal-associated (HMA) domain proteins can give bacteria the ability to resist high metal environments (Maynaud et al., 2014). Rhodanese-Like Domain (RHD) Protein participates in biological processes such as sulfur metabolism and environmental adaptability (Cipollone et al., 2007). Superoxide dismutase (SOD) can effectively catalyze the conversion of superoxide free radicals and protect bacteria from reactive oxygen. It has been shown to be related to the oxidative stress response of *Acinetobacter baumannii* and its resistance to antibiotics (Heindorf et al., 2014). Catalase catalyzes the degradation of hydrogen peroxide and is closely related to the defense of bacteria against related environments (Sun et al., 2016). Universal stress protein (Usp) helps bacteria adapt to oxidative stress, high temperature, pH, etc. (Elhosseiny et al., 2015). Aldehyde dehydrogenase (AldA) is related to a variety of metabolic processes such as redox regulation of bacteria, and can participate in environmental stress defense such as hypochlorite stress (Imber et al., 2018). Cysteine ​​synthase CysK can be used to synthesize cysteine (Bogicevic et al., 2016), and cysteine ​​related products are important molecules required for the oxidative stress response of bacteria (Hicks and Mullholland, 2018). NAD(P)H-quinone oxidoreductase participates in quinone detoxification and helps bacteria to survive under adverse conditions (Ryan et al., 2014), which is also related to resistance to oxidative stress (Kishko et al., 2012). Bacterial proteases play an important role in the survival, stress response, and pathogenicity of bacteria (Culp and Wright, 2017). Isochorismatase family protein is related to serum resistance in *A. baumannii* (Jacobs et al., 2010). Response regulators are related to the tolerance to dehydration and resistance to oxidative stress (Farrow et al., 2018). The toxin antitoxin system also regulates the response of SOS stress (Fernández-García et al., 2016).These proteins all showed upregulated or unique expressed in MDR isolates in this study. The upregulated expression of these stress proteins that resist the external environment may promote the resistance of bacteria by making the resistance of bacteria more stable.

**Table 3:** Stress response-related proteins.

Genbank accession	Uniprot accession	Subcellular localization	Protein name	Classification
KRI51357	A0A0R0RLL9	Unknown	Antibiotic biosynthesis monooxygenase	TMT down-regulated
AFI96375	A0A454AYE3	Unknown	Heat shock protein	TMT up-regulated & Label-free
EXC53193	A0A009SJR6	Cytoplasmic	Trigger factor	TMT up-regulated
ANA38542	A0A0D7TYF2	Cytoplasmic	Chaperone protein DnaK	TMT up-regulated
EXC50791	A0A009T8J5	Unknown	Heavy-metal-associated domain protein	TMT up-regulated
EXC53824	A0A009SLH6	Unknown	Rhodanese-like domain protein	TMT up-regulated
EXA83425	A0A009GB49	Periplasmic	Superoxide dismutase (Fragment)	TMT up-regulated
EXC50244	A0A009SB62	Cytoplasmic	Protein GrpE	TMT up-regulated
PHQ01889	A0A2G1TI69	Cytoplasmic	Catalase	TMT up-regulated
AFI95883	A0A454AX51	Cytoplasmic	Catalase	TMT up-regulated & Label-free
RSR19198	A0A3R9SYU8	Unknown	Universal stress protein (Fragment)	TMT up-regulated
AUT38163	A0A2I8CT30	Cytoplasmic	Aldehyde dehydrogenase	Label-free
EXA83546	A0A009FVR3	Cytoplasmic	Peptidase M16 inactive domain protein	Label-free
ANA36730	A0A0D5YN58	Cytoplasmic	Cysteine synthase	Label-free
POZ07170	A0A0M3FGT4	Cytoplasmic	Chaperone protein HchA	Label-free
OTL46319	A0A241YVG9	Cytoplasmic	NAD(P)H-quinone oxidoreductase (Fragment)	Label-free
AFI94331	A0A454ASQ3	Cytoplasmic	Putative NAD(P)H quinone oxidoreductase, PIG3 family	Label-free
PAM75163	A0A237TK91	Cytoplasmic	Protease	Label-free
SSU69655	A0A335SV40	Periplasmic	Acid shock protein	Label-free
EXB32044	A0A009KMD8	Cytoplasmic	Chaperone protein HscA homolog	Label-free
RSR52999	A0A429MNG9	Unknown	Response regulator (Fragment)	Label-free
KCY73957	A0A062N321	Unknown	Antitoxin	Label-free

### Proteins Related to Gene Expression and Protein Translation

The forth group is proteins that have important functions for gene expression and protein translation or modification (**Table 4**). The expression of Serine hydroxymethyltransferase in this class is down-regulated. This protein is an iron inhibitory protein and can bind to mRNA to control gene expression and participate in the overall bacterial response (Nwugo et al., 2011). Up-regulated proteins includes enolase, DNA breaking-rejoining elements, ribosomal proteins, elongation factor Tu (EF-Tu), ribonuclease E (RNase), Valyl-tRNA synthetase, NusA, and long-chain fatty acid transport proteins. Enolase can play a central role in RNA processing (Krucinska et al., 2019). DNA breaking-rejoining enzymes play an important role in the transmission of genetic elements (Van Houdt et al., 2012). The ribosomal protein S4 RpsD is related to the assembly of ribosomes (Olsson and Isaksson, 1979). The 50S ribosomal proteins L15 and L16 are important translation proteins (McNicholas et al., 2001; Dutton et al., 2016). EF-Tu is also related to protein translation and can interact with a variety of proteins to perform different biological functions (Premkumar et al., 2014). EF-Tu and ribosomal protein can help the production of bacterial protein, and some study have found them upregulated in carbapenem-resistant strains (Tiwari et al., 2012). RNase can perform different processing on RNA to regulate gene expression (Mardle et al., 2019). Valyl-tRNA synthetase is responsible for the aminoacylation of tRNA (Heck and Hatfield, 1988). Nucleoid-associated proteins play an important role in concentrating DNA and regulating gene expression (Lee and Marians, 2013). Transcription termination/antitermination protein NusA can bind to RNA polymerase or nascent RNA to influence transcription (Qayyum et al., 2016). Long-chain fatty acid transport proteins are involved in the transport of fatty acids and can affect intracellular signal transduction and gene expression (Dirusso and Black, 2004).These proteins may help the expression of drug resistance-related proteins by influencing the progress of gene expression or protein translation.

**Table 4:** Proteins related to gene expression and protein translation.

Genbank accession	Uniprot accession	Subcellular localization	Protein name	Classification
EXH77350	A0A140QTL6	Cytoplasmic	Serine hydroxymethyltransferase	TMT down-regulated
PAM68667	A0A334GYE2	Cytoplasmic	Enolase	TMT up-regulated
PAM68199	A0A270N7U1	Unknown	DNA breaking-rejoining protein	TMT up-regulated
EXC51028	A0A009SW10	Cytoplasmic	30S ribosomal protein	TMT up-regulated
WP_001273421	A0A454AZ45	Cytoplasmic	50S ribosomal protein L25	TMT up-regulated
KLT84190	A0A0J0ZQ10	Cytoplasmic	Elongation factor Tu (Fragment)	TMT up-regulated
EXC51023	A0A009SW05	Cytoplasmic	50S ribosomal protein L15	TMT up-regulated
EXC51011	A0A009T933	Cytoplasmic	50S ribosomal protein L16	TMT up-regulated
ANA36598	A0A0B9WR03	Cytoplasmic	Ribonuclease E	Label-free
EXB30595	A0A009KIU0	Cytoplasmic	Valine–tRNA ligase	Label-free
ANA37581	A0A0D8GW25	Unknown	Nucleoid-associated protein	Label-free
WP_000532247	A0A454B0H3	Cytoplasmic	Transcription termination/antitermination protein NusA	Label-free
KHO17349	A0A0B2XPD2	Unknown	Long-chain fatty acid transporter	Label-free
SSS41843	A0A334Z5E6	Unknown	Long-chain fatty acid transport protein	Label-free

### Metabolism-Related Proteins

The largest category contains 60 DEPs expressed differently between the MDR isolates and drug-susceptible isolates. These proteins are mainly related to metabolism (**Supplementary Table 6**). This group includes seven downregulated proteins, which are Serine hydroxymethyltransferase, NADH-quinone oxidoreductase, Malate dehydrogenase, Non-heme chloroperoxidase, 3,4-dihydroxy-2-butanone 4-phosphate synthase, Ketol-acid reductoisomerase [NADP (+)] and Acetyl-coenzyme A carboxylase carboxyl transferase subunit beta. They participate in the biosynthesis of serine, tetrahydrofolate, nitropyrrolidin, branched-chain amino acids, and fatty acids which are related to cellular processes such as bacterial respiration and TCA cycle (Shin et al., 2009; Nwugo et al., 2011; Deris et al., 2014; Reddy et al., 2014; Krishna et al., 2019). Among them, Malate dehydrogenase, which is upregulated in bacterial biofilm state (Shin et al., 2009), and the expression of it in carbapenem-resistant *A.baumannii* was up-regulated, the researcher hypothesis that it contributes to energy production and can improve the survival rate of bacteria (Tiwari et al., 2012). In our results, the protein is down-regulated. We speculate this may be due to strain differences. There are 53 up-regulated or uniquely expressed proteins in MDR strains, which are involved in lipid metabolism (Jang et al., 2008; Gu et al., 2019), amino acid metabolism (Stancik et al., 2002; Bezsudnova et al., 2017), TCA cycle (Corregido et al., 2019), purine anabolic metabolism (Spurr et al., 2012), pyruvate metabolism (Song et al., 2010), fatty acid metabolism (Nishimaki-Mogami et al., 1987), intracellular electron transfer of bacteria (López Rivero et al., 2019), nutrition and energy acquisition (Drewke et al., 1996), and other metabolic processes. In addition, the heavy metal associated (HMA) domain protein which is closely related to the utilization and metabolism of metal ions such as copper ions and zinc ions (Furukawa et al., 2018) is also up-regulated.

### Proteins With Unknown Function or Other Functions Containing Biofilm Formation and Virulence

The final category F includes proteins with unknown functions or other functions besides the other five groups such as virulence or biofilm formation (**Supplementary Table 7**). Studies have found that the resistance of bacteria to disinfectants and antibacterial agents will be greatly increased after the formation of biofilms (Høiby et al., 2010) and the resistance of *A. baumannii* that produces biofilms is significantly higher than that of bacteria that cannot produce biofilms (Gurung et al., 2013). The mechanisms of biofilm formation causing resistance include delaying the penetration of antibacterial agents into bacteria, causing changes in the growth rate of membrane-forming microorganisms, and upregulating efflux pumps and other physiological metabolic differences (Donlan and Costerton, 2002; Kentache et al., 2017). Our study also found the proteins related to biofilm formation expressed more in drug-resistance isolates. For example, flagellin which is involved in the formation of bacterial flagella and the fimbriae assembly protein FilF are both related to bacterial biofilm formation (Karatan and Watnick, 2009). Histidine kinase and esterase members are also involved in the formation of biofilms (Chen et al., 2017; Larsen and Johnson, 2019). They both are upregulated in our study. A comparison of drug-resistant clinical strains and susceptible clinical strains found that drug-resistant clinical strains contain more virulence factors such as FilF, GroEL, and hemagglutinin-like protein (Li et al., 2015). In our study another type of up-regulated protein is mainly related to the virulence of bacteria. For example, hemolysin is one of the virulence factors of bacteria and is closely related to the pathogenicity of bacteria (Bhakdi et al., 1988). Cupin family protein is a superfamily of proteins with multiple functions such as metalloenzymes, sugar binding, and pathogenicity (Sim et al., 2016). Several hypothetical proteins are also up-regulated in MDR strains. Among them, putative septicolysin, cholesterol-dependent cytolysin family, and related proteins are generally virulence factors produced by bacteria (Lukoyanova et al., 2016). Other uncharacterized proteins are also upregulated. It is worth noting that an undefined protein is a member of LysM domain/BON superfamily protein. The unknown functional protein of LysM domain/BON superfamily protein was detected in both upregulated and downregulated proteins. A previous study found that a 16 kDa protein of LysM domain/BON superfamily protein detected in the outer membrane protein of susceptible *Klebsiella pneumoniae* (Kádár et al., 2017). Another study found that it may be related to the stress response of *Klebsiella pneumoniae* to Carbapenem (Khan et al., 2017). However, its specific function is unknown and deserves further study.

### Analysis of Glycoproteomes

Studies have found that O-glycosylation mechanism is widespread in *A.baumannii*, and it is closely related to the virulence and biofilm formation ability of the bacteria (Iwashkiw et al., 2012; Kinsella et al., 2015), but its association with drug resistance was rarely reported. Other post-translational modifications such as phosphorylation and acetylation have been shown to be related to drug resistance (Kentache et al., 2016). Our study aimed to identify the different glycosylation between the MDR and drug-susceptible strains. A total of 77 glycoproteins were found in the 9 MDR isolates and 97 glycoproteins were found in the 10 drug-susceptible isolates. MDR strains had 10 specifically expressed polysaccharides, and drug-susceptible strains had 30 specifically expressed polysaccharides (**Supplementary Table 8**). The specifically expressed polysaccharides found in MDR strain contain chaperone protein DnaK and phosphoenolpyruvate carboxykinase which both are essential for metabolism and survival (**Supplementary Table 9**). By further analysis, we found a polysaccharide form of HexNAc(2)Hex(2)Fuc(1) on the S21 (OGlycan/876.3223) site of adenylate kinase (the product of *adk*) is present in six MDR isolates and not exist in any drug-susceptible isolates. NetOGlyc predicts an additional glycosylation site at site 129 that is more likely to carry O-GalNAc modifications in this protein. The adenylate kinase is related to energy metabolism (Shin and Park, 2015). There is a study showed that the main mechanism of multidrug resistance is the increased activity of adenosine triphosphate (ATP)-dependent drug efflux transporters (Wen et al., 2018). Therefore, we speculate that glycosylation of adenylate kinase is closely related to the metabolism of bacteria, which may enhance the bacteria’s metabolic ability and efflux ability to enhance their drug resistance.

### Transcription Level of 7 DEPs by qRT-PCR

**Figure 6:**
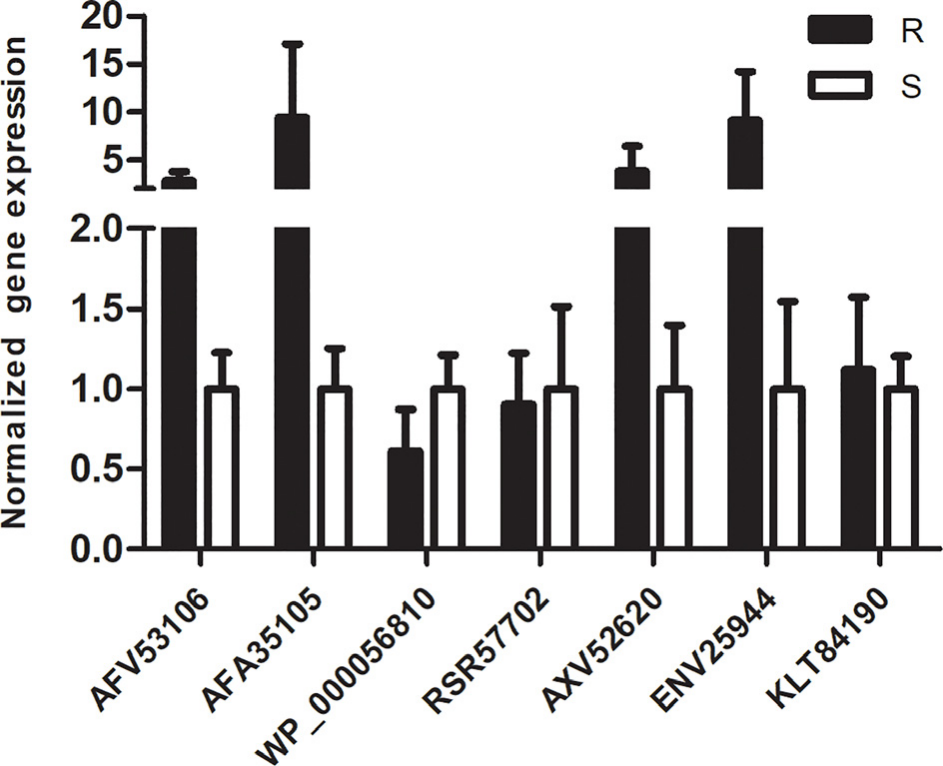
qRT-PCR analysis of 7 representative proteins: The normalized expression level (2-ΔΔct) of 7 DEPs genes of the resistant isolates and susceptible isolates were tested by qRT-PCR. *rpoB* was used as the internal parameter. Error bars represent the SDs.

To verify the results from proteomics, we randomly chose another eight *A.baumannii* isolates from the same hospital to identify the transcription level of 7 DEPs: Aminoglycoside (3’) phosphotransferase AphA1 or APH (3’)-Ia (AFV53106), Beta-lactamase AmpC (AFA35105/AFA35107), Outer membrane protein assembly factor BamD (WP_000056810), MFS transporter (RSR57702), ABC transporter (AXV52620), HlyD membrane-fusion protein of T1SS (ENV25944), and Elongation factor Tu (KLT84190). Our results (**Figure 6**) showed that the transcription levels of AFV53106, AF135105, AXV52620, and ENV25944 in MDR isolates have a higher trend than that of drug-susceptible isolates, which is consistent with the results of proteomics. However, the transcription levels of WP_000056810, RSR57702 and KLT84190 in MDR isolates and susceptible isolates showed no difference. Previous studies have also found disparity between the protein levels and transcription levels of certain genes. This may be due to protein expression and post-translational modifications. In general, the verification of other MDR strains and drug-sensitive strains showed consistency with the proteomic results, indicating that the protein obtained in our results is closely related to the resistance mechanism of *A.baumannii*.

In conclusion, our study found that in MDR strains, a large number of membrane proteins and membrane formation and efflux-related proteins, metabolism-related proteins, stress response-related proteins, and proteins involved in gene expression regulation and protein translation are all upregulated, and glycosylation of adenosine triphosphate is unique in MDR strains. Through the study of the mechanism of multidrug resistance of *A.baumannii*, treatment can be adopted for its resistance mechanism to improve the success rate of treatment of *A.baumannii* infection, such as using engineered endolysin to degrade bacterial peptidoglycan to replace carbapenem drugs (Lee et al., 2017) or add certain compounds that can increase the energy production of bacteria and enhance the permeability of their cell membranes during antibiotic treatment to promote the therapeutic effect of antibiotics (Shin and Park, 2015). The development of vaccines against drug-resistant-related proteins is also another effective strategy to solve drug-resistant bacterial infections (Mujawar et al., 2019). This study uses plenty of isolates for comparative and comprehensive analysis, however, because these isolates were isolated from the same hospital and might have similar genetic phenotypes, we still need further study like expanding sample sources or sequencing these isolates to found the genetic mechanism. In addition, the specific mechanism of how DEPs-related genes influence drug resistance still needs to be further studied and explored using auxiliary methods such as gene knockout to support the results of proteomics.

## Data Availability Statement

The datasets presented in this study can be found in online repositories. The names of the repository/repositories and accession number(s) can be found in the article/**Supplementary Material**.

## Author Contributions

DX and Q-HZ conceived and designed the study. PW and R-QL analyzed the data. LW and W-TY performed experiments. PW and Q-HZ wrote the manuscript. All authors contributed to the article and approved the submitted version.

## Funding

This work was supported by The Major Infectious Diseases AIDS and Viral Hepatitis Prevention and Control Technology Major Projects (Grant No. 2018ZX10712001-006012 and Grand No. 2018ZX10733402-003002).

## Conflict of Interest

The authors declare that the research was conducted in the absence of any commercial or financial relationships that could be construed as a potential conflict of interest.

## Abbreviations

MDR, multidrug-resistant; TMT, tandem mass tag; TEAB, triethyl ammonium bicarbonate; HCD, higher energy collisional dissociation; PPI, protein-protein interaction; DEP, differential expressed proteins; GO, Gene Ontology; MBL, metal β-lactamase; PBP, penicillin binding proteins; BAM, β Barrel Outer Membrane Protein; Omp, Outer membrane protein; RHD, Rhodanese-Like Domain; SOD, Superoxide dismutase; Usp, Universal stress protein.
