# Supplementary figures and images for: Proteomic Analyses of Acinetobacter baumannii Clinical Isolates to Identify Drug Resistant Mechanism
Source: Front Cell Infect Microbiol. 2021 Feb 24;11:625430. doi: 10.3389/fcimb.2021.625430 (PMC7943614; doi:10.3389/fcimb.2021.625430)

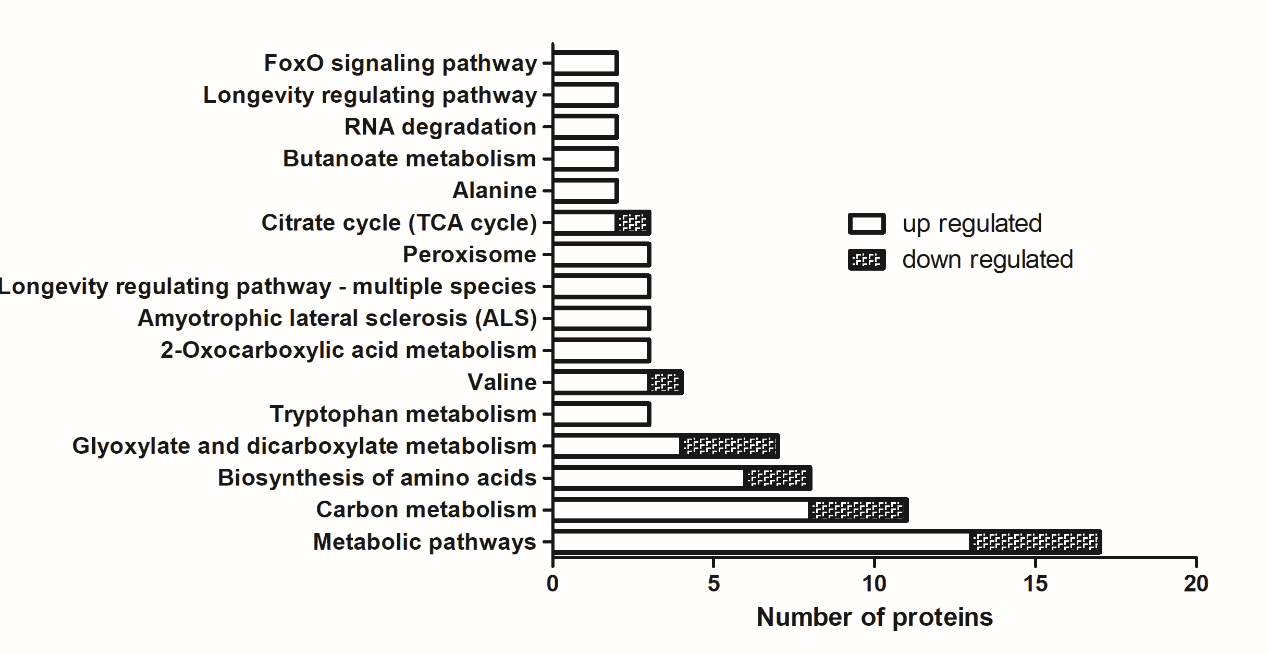


Supplementary Figure 1: The most represented KEGG pathways in TMT-labeling proteomics

Supplement: Supplementary file 1 [file DataSheet_1.zip › Supplementary Figure 1.DOCX]

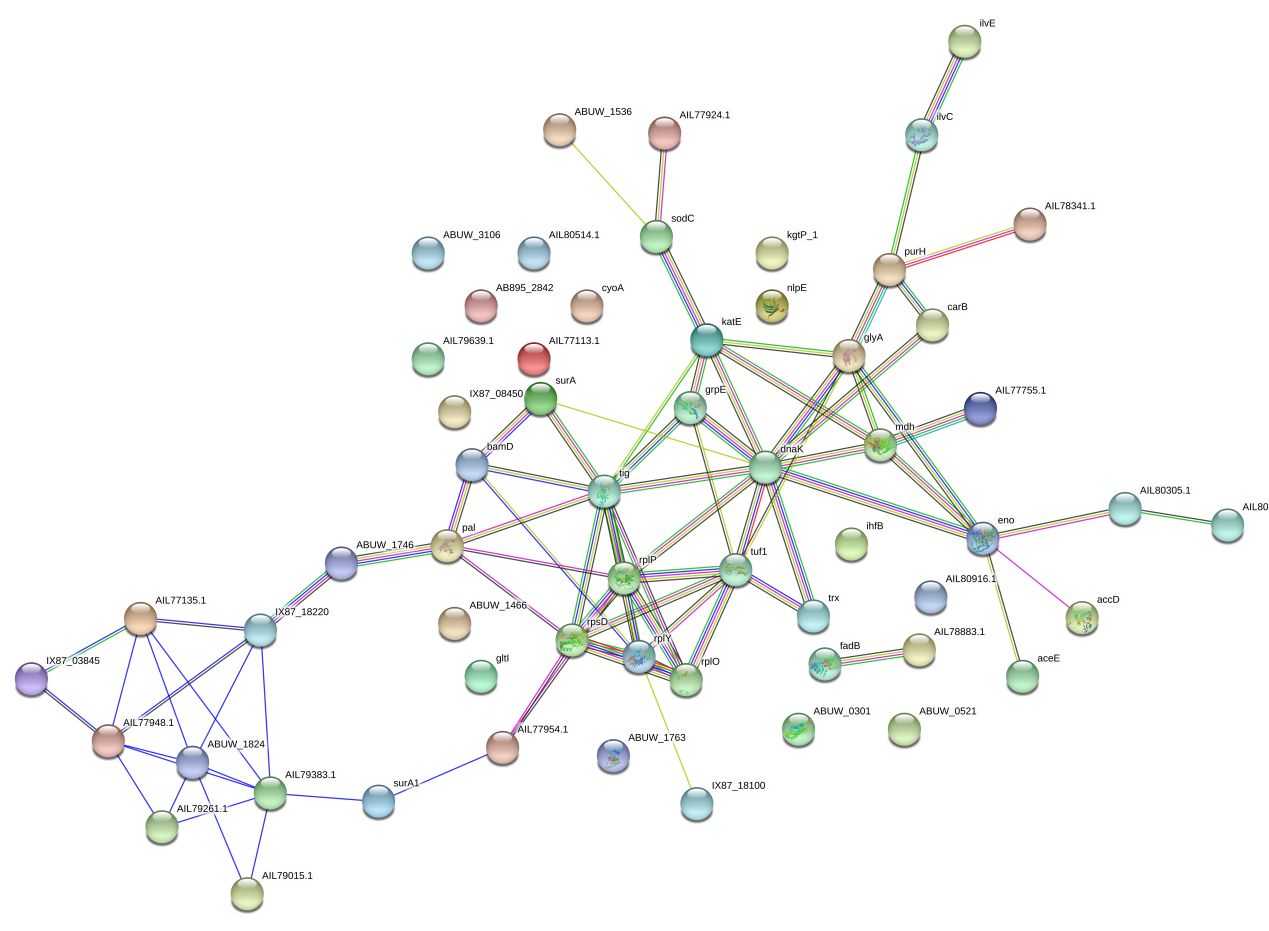


Supplementary Figure 2: Protein protein interaction network of TMT-labeling proteomics

Supplement: Supplementary file 1 [file DataSheet_1.zip › Supplementary Figure 2.DOCX]

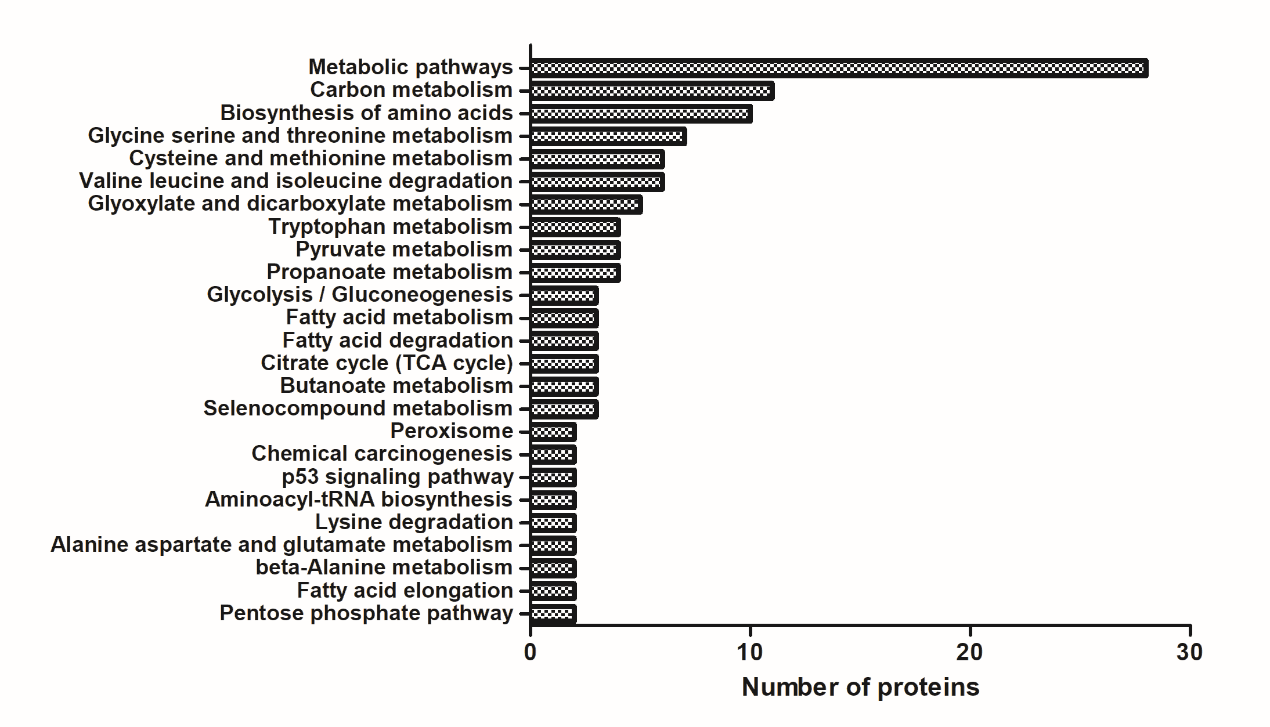


Supplementary Figure 3: The most represented KEGG pathways in Label free proteomics

Supplement: Supplementary file 1 [file DataSheet_1.zip › Supplementary Figure 3.DOCX]

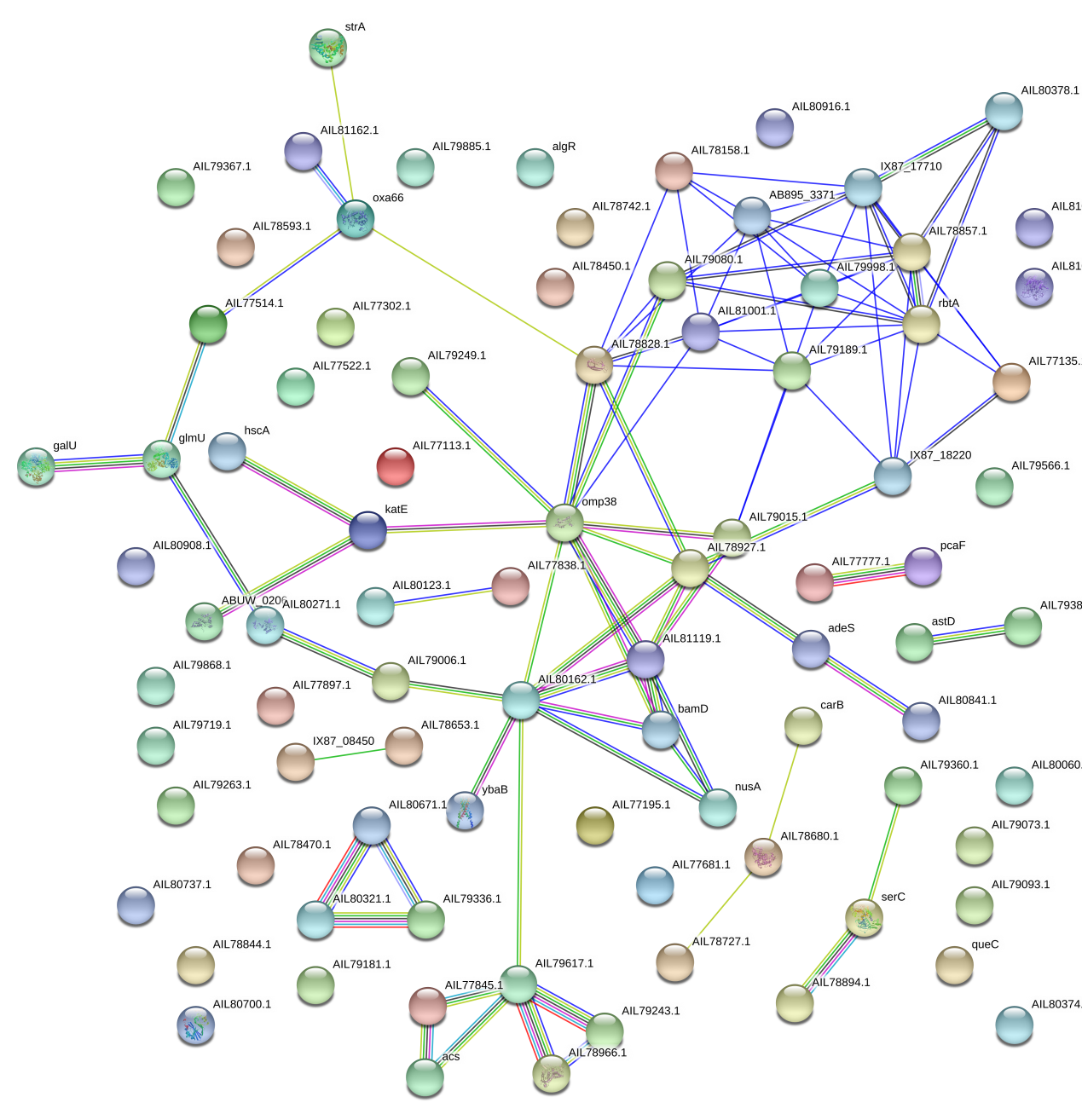


Supplementary Figure 4: Protein protein interaction network of label free proteomics

Supplement: Supplementary file 1 [file DataSheet_1.zip › Supplementary Figure 4.DOCX]
