# Supplementary material for: Proteomic Analyses of Acinetobacter baumannii Clinical Isolates to Identify Drug Resistant Mechanism
Source: Front Cell Infect Microbiol. 2021 Feb 24;11:625430. doi: 10.3389/fcimb.2021.625430 (PMC7943614; doi:10.3389/fcimb.2021.625430)
Supplement: Supplementary file 2 [file DataSheet_2.zip › Supplementary Table 1.DOCX]

**Supplementary Table 1: Primers used in this study**

| Primers | Nucleotide sequence (5′→3′) | The amplified fragment or the utilization |
| --- | --- | --- |
| AFV53106-qF  AFV53106-qR | TCTTCTAATACCTGGAATGC  GTTGCCAATGATGTTACAGA | 155-bp internal fragment of AFV53106, used for qRT-PCR. |
| AFA35105-qF  AFA35105-qR | CACACCGATTGACCAAGTTA  TTGTCTGTATTCACCGATTG | 155-bp internal fragment of AFA35105, used for qRT-PCR. |
| WP_000056810-qF  WP_000056810-qR | AATTATCCGAGCCTTGTCAA  TTGGTTCAGCTTCAGAAGAA | 155-bp internal fragment of WP_000056810, used for qRT-PCR. |
| RSR57702-qF  RSR57702-qR | ATTGTAGTGTTCCGTATTCG  TACCACCAGCAGTTAGGAAT | 155-bp internal fragment of RSR57702, used for qRT-PCR. |
| AXV52620-qF  AXV52620-qR | GGTGCTACTTCTCAAATTCA  GCAAACTGGTTACAAATGTC | 155-bp internal fragment of AXV52620, used for qRT-PCR. |
| ENV25944-qF  ENV25944-qR | CTAAAGTCAGCGTATTAAGC  TGCTCTAACTTCACTACCTT | 155-bp internal fragment of ENV25944, used for qRT-PCR. |
| KLT84190*-*qF  KLT84190-qR | AGCACTTGACTCTTACATCC  CGATCTCTACTTCTTCACCA | 155-bp internal fragment of KLT84190, used for qRT-PCR. |
| *rpoB*-qF  *rpoB*-qR | ATGCCGCCTGAAAAAGTAAC  TCCGCACGTAAAGTAGGAAC | 155-bp internal fragment of *rpoB*, used for qRT-PCR. |
| *16s-PCRF*  *16s-PCRR* | GTGCCAGCAGCCGCGGTAA  GACGGGCGGTGTGTACA | 876-bp internal fragment of 16s rRNA, used for identify the species of isolates. |
